# The association between cystic fibrosis-related diabetes and periodontitis in adults: A pilot cross-sectional study

**DOI:** 10.1371/journal.pone.0305975

**Published:** 2024-06-25

**Authors:** Alaa A. Alkhateeb, Lloyd A. Mancl, Kathleen J. Ramos, Marilynn L. Rothen, Georgios A. Kotsakis, Dace L. Trence, Donald L. Chi

**Affiliations:** 1 Department of Oral Health Sciences, School of Dentistry, University of Washington, Seattle, WA, United States of America; 2 Department of Dental Health Sciences, School of Applied Medical Sciences, King Saud University, Riyadh, Saudi Arabia; 3 Division of Pulmonary, Critical Care, and Sleep Medicine, Department of Medicine, University of Washington, Seattle, WA, United States of America; 4 Department of Periodontics, Dental School, University of Texas Health at San Antonio, San Antonio, TX, United States of America; 5 Department of Global Health, University of Washington, Seattle, WA, United States of America; 6 Division of Metabolism, Endocrinology, and Nutrition, Department of Medicine, University of Washington, Seattle, WA, United States of America; 7 Department of Health Systems and Population Health, School of Public Health, University of Washington, Seattle, WA, United States of America; International Medical University, MALAYSIA

## Abstract

**Objectives:**

Periodontitis is a highly prevalent complication of diabetes. However, the association between cystic fibrosis-related diabetes (CFRD) and periodontitis has not yet been evaluated. The objective of this study was to assess if: 1) CFRD is associated with periodontitis among adults with CF, and 2) periodontitis prevalence differs by CF and diabetes status.

**Methods:**

This was a pilot cross-sectional study of the association between CFRD and periodontitis in adults with cystic fibrosis (CF) (N = 32). Historical non-CF controls (N = 57) from the U.S. National Health and Nutrition Examination Survey (NHANES) dataset were frequency matched to participants with CF on age, sex, diabetes status, and insulin use. We defined periodontitis using the U.S. Centers for Disease Control and Prevention and the American Academy of Periodontology (CDC/AAP) case definition, as the presence of two or more interproximal sites with CAL ≥3 mm and two or more interproximal sites with PD ≥4 mm (not on the same tooth) or one site with PD ≥5 mm. Because NHANES periodontal data were only available for adults ages ≥30 years, our analysis that included non-CF controls focused on this age group (CF N = 19, non-CF N = 57). Based on CF and diabetes status, we formed four groups: CFRD, CF and no diabetes, non-CF with diabetes, and non-CF and no diabetes (healthy). We used the Fisher’s exact test for hypotheses testing.

**Results:**

There was no association between CFRD and periodontitis for participants with CF ages 22–63 years (CFRD 67% vs. CF no diabetes 53%, P = 0.49), this was also true for those ages ≥30 years (CFRD 78% vs. CF no diabetes 60%, P = 0.63). For the two CF groups, the prevalence of periodontitis was significantly higher than for healthy controls (CFRD 78% vs. healthy 7%, P<0.001; CF no diabetes 60% vs. healthy 7%, P = 0.001) and not significantly different than the prevalence for non-CF controls with diabetes (CFRD 78% vs. non-CF with diabetes 56%, P = 0.43; CF no diabetes 60% vs. non-CF with diabetes 56%, P = 0.99).

**Conclusion:**

Among participants with CF, CFRD was not associated with periodontitis. However, regardless of diabetes status, participants with CF had increased prevalence of periodontitis compared to healthy controls.

## Introduction

Cystic fibrosis-related diabetes (CFRD) is a unique type of diabetes mellitus (DM) that shares similarities with major DM types [1, 2]. CFRD affects up to 50% of adults with cystic fibrosis (CF) and adversely impacts the respiratory health, nutritional status, quality of life, and survival of individuals with CF [1, 3, 4]. DM complications like retinopathy and nephropathy occur in CFRD but with less frequency and severity than in other DM types [2].

Periodontitis, defined as the inflammation of hard tissues supporting the teeth, is commonly associated with DM [5]. Potential biological mechanisms linking DM and periodontitis include periodontal bacteria triggering a host defense response, which increases diabetes-induced systemic inflammation and leads to periodontal tissue destruction [6]. The prevalence and severity of periodontitis are both significantly higher in U.S. adults with DM than those without DM [5]. In addition, studies have shown that hyperglycemia (poorly controlled DM) is associated with increased colonization of periodontal bacteria and poorer periodontal treatment outcomes [7, 8]. Despite the strong links between DM and periodontitis [9], there are no studies on whether CFRD has an impact on the periodontal health of adults with CF.

In addition to CFRD, adults with CF present with several additional risk factors for periodontitis, such as increased systemic inflammation because of frequent respiratory infections, inhaled treatments, bone disease, anxiety, and depression [10–12]. It is unknown if CF modifies the association between diabetes and periodontitis. In this study, we tested the following hypotheses: 1) CFRD is associated with periodontitis, and 2) periodontitis prevalence differs by CF and diabetes status.

## Materials and methods

### Study design and study population

This was a cross-sectional pilot study with a historical control group. Adults with CF were recruited from the Adult CF Center at the University of Washington Medical Center, Seattle, U.S.A, from November 2019 to June 2021. We recruited English-speaking adults with CF who were able to provide written informed consent. There were four exclusion criteria. First, to decrease risk of infection for adults with CF, we excluded adults with active infection with *Burkholderia cenocepacia* or *Mycobacterium abscessus* in the prior two years (N = 23). Second, to avoid confounding in the association between diabetes and periodontitis, we excluded adults with pulmonary exacerbation requiring intravenous antibiotics or systemic steroids in the prior 4 weeks (N = 15). Third, smoking is a known risk factor for periodontitis. Because smoking is rare among adults with CF, we excluded adults with a history of smoking (N = 7). Fourth, we excluded adults with CF with no history of an Oral Glucose Tolerance Test (OGTT) in the last two years (N = 41). Finally, to avoid risk of endocarditis, we excluded adults who needed prophylactic antibiotics for dental visits (N = 4) (S1 Fig in S1 File).

A historical control group without CF was identified from the 2013–2014 U.S. National Health and Nutrition Examination Survey (NHANES) dataset which included periodontal examination data for participants ages ≥30 years. Thus, our control group consisted of individuals from this age group. NHANES participants were included if they had complete documentation of periodontal examinations, laboratory tests for diabetes, medical history questions for diabetes, and interviews assessing demographics (S2 Fig in S1 File). We excluded NHANES participants with impaired glucose tolerance and smokers. NHANES does not include a variable for CF. Thus, to avoid including adults with CF from NHANES in our control groups, we excluded NHANES participants who reported chronic obstructive respiratory diseases and chronic bronchitis. Non-CF controls were frequency matched three to one to participants with CF on age, sex, diabetes status, and insulin use.

We collected identifiable data from participants (e.g., medical record numbers). These data were saved in encrypted password-protected files and only the primary investigator had access to these data. The data were de-identified after completing the study. The study was approved by the Institutional Review Board at the University of Washington (protocol number: 00007976).

### Recruitment of participants with CF

We obtained a HIPAA waiver of consent from the Institutional Review Board at the University of Washington to allow for a medical record screening to identify eligible adults with CF. Adults with CF were recruited by phone, email, or in person during their routine CF clinic visits. We scheduled a two-hour study visit at the dental research clinic at the University of Washington School of Dentistry. We followed the Cystic Fibrosis Foundation infection control protocols during the study visit. We obtained a written informed consent and HIPAA authorization from participating adults with CF at the study visit to allow for subsequent abstraction of medical record data. Informed consent and HIPAA authorization were documented using RedCap™. The informed consent procedure was approved by the Institutional Review Board at the University of Washington. Participants with CF with unmet dental treatment needs were provided a referral to the University of Washington dental clinics. We provided oral hygiene instructions, an oral hygiene kit with a toothbrush and fluoridated toothpaste, and $95 as reimbursement for transportation and compensation for the participant’s time.

There were four study procedures: 1) survey, 2) periodontal screening, 3) medical record abstraction, and 4) control data procurement.

#### Survey

We designed a 17-item survey (**S1 Table in** **S1 File**). The survey included questions on sociodemographic factors (race, ethnicity, household income, education level, dental insurance), history of dental care utilization, and personal oral hygiene habits. We designed the survey using REDCap™ [13] and participants used a study iPad to complete the survey.

#### Periodontal screening

Each participant received a full-mouth periodontal screening conducted by a trained and calibrated registered dental hygienist [14]. The periodontal screening included a full-mouth assessment of periodontal pocket depth (PD) and gingival margin level using a manual UNC-15 periodontal probe. These two periodontal measures were assessed at six sites per tooth for all teeth present, excluding third molars [15]. PD was defined as the distance in millimeters (mm) from the free gingival margin to the bottom of periodontal sulcus. Gingival margin level was defined as the distance in mm from the free gingival margin to the cementoenamel junction. Clinical attachment loss (CAL) was calculated by subtracting gingival margin level from PD using the following equation: CAL = PD–(+/–) gingival margin level. All measurements were rounded to the lowest whole mm [15].

#### Medical record abstraction for participants with CF

For participants with CF, we abstracted sociodemographic and medical data from electronic medical records to generate study variables. Data included age, sex, health insurance type, CF-transmembrane conductance regulator (CFTR) genotype, most recent forced expiratory volume in one second percent predicted (FEV_1_% predicted), any previous referral for lung transplant evaluation, CFRD status, glycated hemoglobin (HbA1c), body mass index (BMI), and CFTR modulator therapy use. CFTR modulator therapy was defined as triple CFTR modulator therapy (elexacaftor/tezacaftor/ivacaftor), other CFTR modulator therapy (ivacaftor, lumacaftor/ ivacaftor, or tezacaftor/ ivacaftor), or none.

#### Control data procurement

For non-CF controls in the 2013–2014 NHANES, we obtained sociodemographic data (age, sex, race, ethnicity, income level, education level), medical data (diabetes status, HbA1c, BMI), and periodontal measures (PD, CAL). Periodontal measures were collected from NHANES using the same protocol used for participants with CF described above [16].

### Outcome measure

Our outcome was periodontitis [yes/no]. We defined periodontitis using the U.S. Centers for Disease Control and Prevention and the American Academy of Periodontology (CDC/AAP) case definition [17], as the presence of two or more interproximal sites with CAL ≥3 mm and two or more interproximal sites with PD ≥4 mm (not on the same tooth) or one site with PD ≥5 mm.

### Comparison groups

Participants with CF were divided into two groups (those with CFRD, and all others). CFRD was defined based on an established diagnosis by a physician following the American Diabetes Association diagnostic criteria using an OGTT of 200 mg/dL or greater or HbA1c greater than 6.5%. All others had a normal OGTT within the last two years. Non-CF controls were also divided into two groups (non-CF with diabetes and non-CF without diabetes), with diabetes status defined based on self-reported diabetes and HbA1c. Thus, there were four comparison groups: CFRD, CF and no diabetes, non-CF with diabetes, and non-CF with no diabetes (healthy).

### Data analysis

We generated descriptive statistics and reported means and standard deviations (SD) for normally distributed quantitative variables, medians and interquartile ranges (IQR) for non-normally distributed quantitative variables, and frequencies and percentages for categorical variables. We presented descriptive data for all participants with CF and for participants with CF divided into CFRD status (yes/no). We also presented descriptive data for study groups with and without CF ages 30 years and older grouped by diabetes status. To test for differences in the study variables, we used the independent samples t-test for normally distributed quantitative variables, the Mann-Whitney test for non-normally distributed quantitative variables, the Fisher’s exact test for binary variables, and the exact chi-square test for categorical variables.

For hypothesis testing, first, we used the Fisher’s exact test to assess the association between CFRD and periodontitis for all enrolled participants with CF ages 22–63 years. Second, we used the independent samples t-test to assess differences in means of clinical periodontal measures (PD, CAL) between participants with CF grouped by CFRD status. Third, we used the Fisher’s exact test to compare the prevalence of periodontitis in participants ages 30 years and older across the four comparison groups (CFRD, CF and no diabetes, non-CF with diabetes, and healthy). Fourth, we used the Welch’s analysis of variance with the Games-Howell post hoc test to assess for between groups differences in PD and CAL for the four comparison groups and adjusted for multiple testing. We reported the difference in means and 95% confidence intervals (CI).

Alpha was set at 0.05 and all tests were two-sided. Data were analyzed using Statistical Package for the Social Sciences (SPSS) 19.0 for Mac.

## Results

### Participants with CF by diabetes status

Participants with CF with and without diabetes did not differ in sociodemographic characteristics (**Table 1**). Compared to participants in the CF group without diabetes, those with CFRD had a higher median HbA1c (median: CFRD 6.2% vs. CF no diabetes 5.4%, P<0.001). There were no other differences in other medical and behavioral characteristics between the two CF groups.

**Table 1:** Sociodemographic, medical, and dental characteristics of adults with CF grouped by CFRD status (N = 32).

	Overall N = 32	Cystic Fibrosis-Related Diabetes		
		Yes N = 15	No N = 17	P-value ^a^
**Sociodemographic characteristics**				
**Age (years) (Median (IQR))**	30.0 (28.0–38.0)	30.0 (28.0–39.0)	30.0 (28.5–32.5)	0.86
**Sex** (**N (%))**				0.29
Female	20 (62.5)	11 (73.3)	8 (47.1)	
Male	12 (37.5)	4 (26.7)	9 (52.9)	
**Race** (**N (%))**				0.47
Black or African American	1 (3.1)	1 (6.7)	0 (0.0)	
White	31 (96.9)	14 (93.3)	15 (100.0)	
**Ethnicity** (**N (%))**				1.0
Hispanic/Latino	2 (6.3)	1 (6.7)	1 (5.9)	
**Health Insurance** (**N (%))**				0.42
Public	8 (25.0)	5 (33.3)	3 (17.6)	
Private or dual	24 (75.0)	10 (66.7)	14 (82.4)	
**Annual household income (US$) (N (%))**				1.0
<$70,000	8 (27.6)	4 (30.8)	4 (25.0)	
≥$70,000	21 (72.4)	9 (69.2)	12 (75.0)	
Declined to answer	3	2	1	
**Education level** (**N (%))**				0.30
High school, GED, or equivalent	4 (12.5)	3 (20.0)	1 (5.9)	
Some college	7 (21.9)	3 (20.0)	4 (23.5)	
4-year college degree	11 (34.4)	3 (20.0)	8 (47.1)	
More than 4-year college degree	10 (31.3)	6 (40.0)	4 (23.5)	
**Dental insurance** (**N (%))**	29 (90.6)	12 (80.0)	17 (100.0)	0.09
**Medical characteristics**				
**CF genotypes** (**N (%))**				0.76
F508del homozygous	13 (40.6)	7 (46.7)	6 (35.3)	
F508del heterozygous	15 (46.9)	6 (40.0)	9 (52.9)	
Other mutations	4 (12.5)	2 (13.3)	2 (11.8)	
**FEV1% predicted (mean ± SD)**	74.1±21.9	71.2±20.8	76.7±23.2	0.25
**Referred for lung transplant (N (%))**	1 (3.1)	1 (7.1)	0 (0.0)	0.33
**Hemoglobin A1c (median (IQR))**	5.6 (5.4–6.2)	6.2 (5.9–7.3)	5.4 (5.3–5.6)	**<0.001**
**Body Mass Index (kg/m** ^**2**^ **) (median (IQR))**	23.1 (21.1–27.4)	22.9 (20.5–26.9)	23.3 (21.4–28.1)	0.62
**CFTR modulator therapy** (**N (%))**				0.31
Elexacaftor/tezacaftor/ivacaftor	18 (56.3)	9 (60.0)	9 (52.9)	
Other modulators therapies ^b^	10 (31.3)	3 (20.0)	7 (41.2)	
None	4 (12.5)	3 (20.0)	1 (5.9)	
**Dental characteristics** (**N (%))**				
**Last dental visit** ^c^				0.15
Less than one year	24 (75.0)	10 (66.7)	14 (82.4)	
One year or more	5 (15.6)	2 (13.3)	3 (17.6)	
Three years or more	3 (9.4)	3 (20.0)	0 (0.0)	
**Unable to get needed dental treatment** ^c^	8 (25.0)	5 (33.3)	3 (17.6)	0.42
**Tooth brushing frequency** ^c^				0.76
Less than once a day	4 (12.5)	2 (13.3)	2 (11.8)	
Once a day	13 (40.6)	7 (46.7)	6 (35.3)	
Two or more times a day	15 (46.9)	6 (40.0)	9 (52.9)	
**Daily flossing** ^c^	8 (25.0)	5 (33.3)	3 (17.6)	0.42

^a^ Independent samples t-test was used for normally distributed continuous variables, Mann-Whitney test for skewed continuous variables, Fisher exact test for binary variables, and exact chi square test for categorical variables.

^b^ Other CFTR modulator therapy = ivacaftor, lumacaftor/ ivacaftor, or tezacaftor/ ivacaftor

^c^ Self-reported

P-Value < 0.05 is **bolded**

### Non-CF controls

Compared to non-CF controls, the CF group ages 30 years and older had a higher proportion of white participants (CF 100% vs. non-CF 67%, P = 0.038) (**Table 2 and S2 Table in** **S1 File**); a lower proportion of participants with an annual income of less than $70,000 (CF 17% vs. non-CF 52%, P = 0.013); and a lower median BMI (CF 23.4 kg/m^2^ vs. non-CF 28.4 kg/m^2^, P = 0.002).

**Table 2:** Sociodemographic and medical characteristics of adults with CF and 2013–2014 NHANES non-CF controls ages 30 years and older grouped by CF status and diabetes status (N = 76).

	Overall		
	CF (N = 19)	Non-CF (N = 57)	P-value ^a^
**Age (years) (Median (IQR))**	33.0 (30.0–52.0)	38.0 (31.0–47.0)	0.51
**Sex** (**N (%))**			1.0
Female	11 (57.9)	33 (57.9)	
Male	8 (42.1)	24 (42.1)	
**Race** (**N (%))**			**0.038**
Mexican American	0 (0.0)	10 (17.5)	
White	19 (100)	38 (66.7)	
Other /multiracial	0 (0.0)	9 (15.8)	
**Ethnicity** (**N (%))**			0.27
Hispanic/Latino	1 (5.3)	11 (19.3)	
**Annual household income (US$) (N (%))**			**0.013**
<$70,000	3 (16.7)	28 (51.9)	
≥$70,000	13 (83.3)	26 (48.1)	
Declined to answer or missing	1	3	
**Education level** (**N (%))**			0.15
Less than high school	0 (0.0)	9 (15.8)	
High school, GED, or equivalent	1 (5.3)	6 (10.5)	
Some college	6 (31.6)	20 (35.1)	
4-year college degree or more	12 (63.1)	22 (38.6)	
**Body Mass Index (kg/m** ^**2**^ **) (Median (IQR))**	23.4 (21.5–26.9)	28.4 (25.7–35.2)	**0.002**
**Hemoglobin A1c (Median (IQR))**	5.7 (5.4–6.2)	5.5 (5.1–7.5)	0.78

^a^ The Mann-Whitney test for skewed continuous variables, Fisher exact test for binary variables, and exact chi square test for other categorical variables.

P-Value < 0.05 is **bolded**

### Outcome measure

#### Periodontitis and CFRD in participants with CF

CFRD was not associated with periodontitis (CFRD 67% vs. CF no diabetes 53%, P = 0.49) (**Table 3**). Participants with CF with and without diabetes had similar means of clinical periodontal measures (PD: CFRD 2.4±0.11 mm vs. CF no diabetes 2.4±0.13 mm, P = 0.58), (CAL: CFRD 1.8±0.33 mm vs. CF no diabetes 1.7±0.28 mm, P = 0.18).

**Table 3:** Comparison of prevalence of periodontitis and means of clinical periodontal measures of adults with CF grouped by CFRD status (N = 32).

	Overall N = 32	CFRD		
		Yes N = 15	No N = 17	
	N (%)	N (%)	N (%)	P-value ^a^
**Periodontitis N (%)**	19 (59.4)	10 (66.7)	9 (52.9)	0.49
**Periodontal measure**	**Mean ±SD**	**Mean ±SD**	**Mean ±SD**	**P-value** ^b^
Periodontal pocket depth (mm)	2.4±0.14	2.4±0.11	2.4±0.13	0.58
Clinical attachment loss (mm)	1.7±0.30	1.8±0.33	1.7±0.28	0.18

^a^ Fisher’s exact test

^b^ Independent samples t-test

#### Periodontitis and CF-Diabetes status

The prevalence of periodontitis for the two CF groups, with and without diabetes, ages 30 years and older were significantly higher than the prevalence of periodontitis for the healthy control group (CFRD 78% vs. healthy 7%, P<0.001; CF no diabetes 60% vs. healthy 7%, P = 0.001) (**Table 4**) and not significantly different than the prevalence of periodontitis for the non-CF control group with diabetes (CFRD 78% vs. non-CF with diabetes 56%, P = 0.43; CF no diabetes 60% vs. non-CF with diabetes 56%, P = 0.99).

**Table 4:** Prevalence of periodontitis for adults with CF ages 30 years and older grouped by CFRD status compared to non-CF controls with and without diabetes (N = 76).

CF-Diabetes group	Periodontitis	
	%	P-value ^a^
**CFRD vs. healthy**	77.8% vs. 6.7%	**<0.001**
**CF no diabetes vs. healthy**	60.0% vs. 6.7%	**0.001**
**Non-CF with diabetes vs. healthy**	55.6% vs. 6.7%	**<0.001**
**CFRD vs. non-CF with diabetes**	77.8% vs. 55.6%	0.43
**CF no diabetes vs. non-CF with diabetes**	60.0% vs. 55.6%	1.0

^a^ Fisher’s exact test.

Healthy = no CF and no diabetes

Compared to healthy controls, both CF groups had significantly deeper PD (CFRD vs. healthy: mean difference = 1.3, 95CI% = 1.1,1.5, P<0.001; CF no diabetes vs. healthy: mean difference = 1.3, 95CI% = 1.1,1.5, P<0.001), and significantly greater CAL (CFRD vs. healthy: mean difference = 0.66, 95CI% = 0.30,1.0, P<0.001; CF no diabetes vs. healthy: mean difference = 0.51, 95CI% = 0.23,0.80, P<0.001) (**Table 5**). The two CF groups had significantly deeper PD than non-CF controls with diabetes (CFRD vs. non-CF with diabetes: mean difference = 0.91, 95CI% = 0.60,1.2, P<0.001; CF no diabetes vs. non-CF with diabetes: mean difference = 0.90, 95CI% = 0.58,1.2, P<0.001), but similar CAL (CFRD vs. non-CF with diabetes: mean difference = 0.09, 95CI% = -0.66,0.52; P = 0.97; CF no diabetes vs. non-CF with diabetes: mean difference = -0.06, 95CI% = -0.62,0.50, P = 0.99).

**Table 5:** Difference in means of clinical periodontal measures for adults with CF ages 30 years and older grouped by CFRD status compared to non-CF controls with and without diabetes (N = 76).

CF-Diabetes group	Periodontal pocket depth		Clinical attachment loss	
	Difference (95% CI)	P-value ^a^	Difference (95% CI)	P-value ^a^
**CFRD vs. healthy**	1.3 (1.1,1.5)	**<0.001**	0.66 (0.30,1.0)	**<0.001**
**CF no diabetes vs. healthy**	1.3 (1.1,1.5)	**<0.001**	0.51 (0.23,0.80)	**<0.001**
**Non-CF with diabetes vs. healthy**	0.39 (0.06,0.71)	**0.014**	0.57 (0.04,1.1)	**0.029**
**CFRD vs. non-CF with diabetes**	0.91 (0.60,1.2)	**<0.001**	0.09 (-0.50,0.67)	0.97
**CF no diabetes vs. non-CF with diabetes**	0.90 (0.58,1.2)	**<0.001**	- 0.06(-0.62,0.50)	0.99

^a^ Games-Howell post hoc test

Healthy = no CF and no diabetes

P-Value < 0.05 is **bolded**

## Discussion

To our knowledge, this is the first study to assess the association between CFRD and periodontitis and to include non-CF controls in the assessment of periodontal health of adults with CF. There were two main findings. First, the prevalence of periodontitis did not differ by CFRD status among participants with CF. Second, both CF groups (with and without diabetes) ages 30 years and older had higher prevalence of periodontitis than healthy controls and similar prevalence of periodontitis to non-CF controls with diabetes, despite adults with CF having higher socioeconomic status than non-CF controls.

First, CFRD was not associated with periodontitis among participants with CF in our study. This finding is inconsistent with studies of non-CF adults with DM that showed increased prevalence and severity of periodontitis in DM [5, 18]. There are three potential interconnected explanations for our findings. First, periodontitis is associated with poorly controlled diabetes. Studies have shown that the prevalence and severity of periodontitis increased with HbA1c values of 8.0% and higher [19]. Overall, our CFRD group had well-controlled diabetes. Only two participants with CFRD had an HbA1c value higher than 8.0% and two-thirds of participants with CFRD had HbA1c of less than 6.5%. Second, temporary hyperglycemia occurs in the absence of CFRD for adults with CF, which is mainly related to steroid treatment of pulmonary exacerbations [1, 4, 20]. Studies of non-CF adults showed that prediabetes, indicated by glucose level that is higher than normal but lower than diabetes level, was associated with increased periodontitis [8]. Fluctuations in blood glucose levels in adults with CF without diabetes might have mitigated the difference in periodontitis by CFRD status in our study. Third, most of our study participants were white, had relatively high socioeconomic status, had dental insurance, maintained optimal oral hygiene and reported frequent utilization of preventive dentistry, all of which are factors that are protective against periodontitis [18, 21]. Future studies of CFRD and periodontal health should include a more diverse population with CF including non-whites, lower socioeconomic status, and more severe CFRD. Collecting longitudinal data could help account for the impact of steroid use and episodes of hyperglycemia.

Second, regardless of their CFRD status, participants with CF had higher prevalence of periodontitis than healthy controls and similar prevalence of periodontitis to non-CF controls with diabetes, despite having higher socioeconomic status than non-CF controls. There are two potential explanations. First, adults with CF without diabetes are at higher risk for prediabetes than non-CF adults without diabetes [20], which potentially increased their risk for periodontitis. Participants with CF without diabetes in our study had significantly higher median HbA1c values than healthy controls (**S3 Table in** **S1 File**). Increased HbA1c is independently associated with increased periodontitis in the general population [5]. However, increased HbA1c by itself does not fully explain the difference in the prevalence of periodontitis in our study, as participants with CF without diabetes had a significantly lower HbA1c than participants with CFRD who had a significantly lower HbA1c than non-CF controls with diabetes, yet the three groups had similar prevalence of periodontitis. Another potential explanation is that CF modifies the risk of periodontitis. The impact of increased HbA1c on the periodontal health for participants with CF might have been amplified by other risk factors for periodontitis like increased systemic inflammation associated with frequent lung infections, frequent use of inhaled medications, and the presence of other comorbidities like asthma, bone disease, anxiety, and depression [10–12, 22]. Larger longitudinal studies with concurrent controls are needed to measure the impact of glucose fluctuation and confirm if CF modifies the risk of periodontitis as suggested by our findings.

Our study showed that regardless of their CFRD status participants with CF are at similar risk of periodontitis as non-CF adults with diabetes, a well-defined high-risk group for periodontitis. This is particularly concerning for adults with CF because periodontitis is linked to poor diabetes control and increased systemic inflammation, both of which are risk factors for poor CF outcomes [23–25]. In addition, studies have showed risk of micro-aspiration of oral bacteria and links between oral infections and decreased lung function in individuals with lung diseases [26–28].

There are two important considerations in the interpretation of periodontitis prevalence by CF status in our study. First, while individuals in the two CF groups had a higher prevalence of periodontitis than healthy controls, individuals in all three groups had mild to moderate periodontitis (**S3 Table in** **S1 File**). Second, while the non-CF controls were from a nationally representative data source, individuals in the non-CF groups are not representative of all adults enrolled in NHANES. For example, the non-CF groups did not include older adults (≥65 years) and adults with a history of smoking, two population subgroups at increased risk for periodontitis. According to the latest NHANES analyses, about one-half of the U.S. population had periodontitis with increased risk among smokers, individuals with obesity, and adults with severe diabetes, most of whom are not likely to be represented in our non-CF control groups [5, 29, 30].

Collectively, our findings call for strategies to ensure optimal oral health for adults with CF. Strategies should focus on increasing oral health awareness and facilitating dental care access, as well as conducting further clinical research. First, oral health awareness for adults with CF can be improved by integrating oral health promotion as part of the CF care during clinic visits and at community level. Routine CF care should include promotion of oral health by conducting regular dental screenings and discussions of the importance of optimal oral health to the maintenance of general health and quality of life. CF advocacy groups have demonstrated successes in promoting health related practices for individuals with CF [31]. These organizations can partner with dentists and dental hygienists to promote efforts to build resources and provide education to meet the oral health needs of adults with CF. In hospital settings with dental clinics, pairing dental visits with the routine CF care visits might be an efficient way to increase dental care utilization for adults with CF. As part of dental care, behavioral interventions like motivational interviewing could be utilized with the goal of defining barriers to optimal oral health and ways to mitigate the potential impact of CF and related treatments on oral health. Finally, the current literature is inadequate to generate evidence-based dental care recommendations specific for adults with CF. There is a need for longitudinal multicenter studies with a representative sample of all adults with CF to build a framework of CF-related risk factors for dental diseases that would guide clinical care recommendations.

There were four main limitations to our study. First, the study had limited power because of small sample size. Recruitment and clinical data collection were adversely impacted by the COVID-19 pandemic. Second, the time of diabetes screening for adults without CFRD was not uniform in our study. We identified adults with CF without diabetes based on a normal OGTT result from the past two years. Episodes of glucose abnormality are common in adults with CF without diabetes, proposing a potential confounder in the association between diabetes and periodontitis that we did not account for in this study [20]. We opted for the two-year time for an OGTT due to the low adherence to annual OGTT that has been demonstrated in the CF population [32]. Of the 90 individuals excluded from our study, 41 individuals did not have OGTT in the last two years. In addition, adults with CF who had completed the recommended regular OGTT, and as a result were eligible to participate in our study, were likely to be different in terms of behavioral factors than those who did not adhere to the recommended regular OGTT. This biased our study population toward a CF population with high adherence to health-related behaviors which may have also been reflected in their periodontal health status. It is possible that rates of periodontitis are higher among those individuals who have not undergone routine OGTT screening. Future studies should use a standardized approach to define CF adults without diabetes, potentially with continuous glucose monitoring at the time of dental evaluation. Third, to avoid confounding in the association between diabetes and periodontitis, we excluded individuals with CF who were treated with steroids for pulmonary exacerbations in the last 4 weeks. Thus, our study did not include individuals with advanced CF disease who experience frequent pulmonary exacerbations which biased our study population toward a healthier CF population. Fourth, we conducted our study at a single site, and we had a historical control group. Most of our study population was white, high-income, and had dental insurance, all of which are factors associated with a lower risk of periodontitis [18]. Future studies should focus on recruiting a more diverse study population, especially across race, ethnicity, and income.

## Conclusion

In conclusion, among adults with CF, CFRD was not associated with periodontitis. However, CF is a potential risk factor for periodontitis as our study groups with CF had higher prevalence of periodontitis than healthy controls and similar prevalence of periodontitis as non-CF controls with diabetes–a well-defined high-risk group for periodontitis. There is a need for larger, multicenter, longitudinal studies with concurrent control group to understand how CF-related comorbidities and treatments impact the periodontal health of adults with CF. These data could be used to update dental care recommendations for adults with CF and to develop interventions aimed at improving the oral and systemic health and the quality of life of this vulnerable population.

## Supporting information

S1 Checklist PLOS ONE Alkhateeb STROBE-checklist-cross-sectional. (DOC)

S1 File (PDF)
