# Supplementary material for: The association between cystic fibrosis-related diabetes and periodontitis in adults: A pilot cross-sectional study
Source: PLoS One. 2024 Jun 25;19(6):e0305975. doi: 10.1371/journal.pone.0305975 (PMC11198763; doi:10.1371/journal.pone.0305975)

Supporting information

S1 Table. Oral Health survey used to collect sociodemographic and behavioral information from participants with CF

Survey question	Answer choices
Part 1: Oral Health	
1. Overall, how would you rate the health of your teeth and gums?	- Excellent<br>- Very good<br>- Good<br>- Fair<br>- Poor<br>- I don't know
2. Which of the following types of home care products do you use? (Check all that apply)	- Regular toothbrush<br>- Electric toothbrush<br>- Floss<br>- Interdental brush<br>- Waterpik<br>- Other
3. How often do you brush your teeth?	- More than once a day<br>- Once a day<br>- A few times a week<br>- Once a week<br>- Never<br>- I don't know

4. How often do your teeth or gums bleed when they are brushed?	- Always<br>- Sometimes<br>- Never<br>- I don't know
5. Do you floss your teeth?	- Yes<br>- No
6. How often do you floss your teeth?	- More than once a day<br>- Once a day<br>- A few times a week<br>- Once a week<br>- I don't know
7. How often do your teeth or gums bleed when you use dental floss?	- Never<br>- Sometimes<br>- Always
8. Have you ever used tobacco?	- Yes<br>- No
9. Do you have dental insurance?	- Yes<br>- No
10. When was the last time you visited a dentist?	- 6 months ago or less<br>- More than 6 months, but not more than 1 year ago<br>- More than 1 year ago<br>- More than 3 years ago<br>- I have never been to the dentist

11. During the past 12 months, was there a time when you needed dental care but could not get it at that time?	- Yes<br>- No
Part 2: Miscellaneous Questions	
12. What is your date of birth	- mm/dd/yyyy
13. What is your race/ethnicity? (Check all that apply)	- American Indian or Alaska Native<br>Asian<br>- Black or African American<br>- Native Hawaiian or Pacific Islander<br>- White or Caucasian<br>- Other
14. Are you Hispanic or Latino(a)?	- Yes<br>- No
15. What is the highest level of schooling you completed?	- Less than high school<br>- High school, GED, or equivalent<br>- Some college<br>- 4-year college degree<br>- More than 4-year college degree
16. Do you receive assistance to pay for food (like SNAP or food stamps)?	- Yes<br>- No
17. What is the estimated total annual income of all people in your household?	- Less than $20,000<br>- $20,000 to $29,999<br>- $30,000 to $49,999<br>- $50,000 to $69,999

	- $70,000 to $89,999<br>- More than $90,000<br>- I don't know<br>- I prefer not to answer

S2 Table. Sociodemographic and medical characteristics of adults with CF and 2013-2014 NHANES non-CF controls ages 30 years and older grouped by CF and diabetes status (N=76).

	Diabetes					
	Yes			No		
	CF (N=9)	Non-CF (N=27)	P-value [a]	CF (N=10)	Non-CF (N=30)	P-value [a]
**Age (years) (Median (IQR))**	39.0 (30.5-53.0)	42.0 (38.0-50.0)	0.28	31.5 (30.0-45.5)	31.0 (30.0-35.0)	0.89
**Sex (N (%))**			1.0			1.0
Female	7 (77.8)	21 (77.8)		4 (40.0)	12 (40.0)	
Male	2 (22.2)	6 (22.2)		6 (60.0)	18 (60.0)	
**Race (N (%))**			0.08			0.38
Mexican American	0 (0.0)	7 (25.9)		0 (0.0)	3 (10.0)	
White	9 (100.0)	14 (51.9)		10 (100.0)	24 (80.0)	
Other /multiracial	0 (0.0)	6 (22.2)		0 (0.0)	3 (10.0)	
**Ethnicity (N (%))**			0.40			0.56
Hispanic/Latino	1 (11.1)	8 (29.6)		0 (0.0)	3 (10.0)	
**Annual household income (US$)**			0.053			0.14
**(N (%))**	1 (12.5)	14 (53.8)		2 (20.0)	14 (50.0)	
<$70,000	7 (87.5)	12 (46.2)		8 (80.0)	14 (50.0)	
≥$70,000	1	1		0	2	
Declined to answer or missing						
**Education level (N (%))**			**0.006**			0.85
Less than high school	0 (0.0)	7 (25.9)		0 (0.0)	2 (6.7)	
High school, GED, or equivalent	0 (0.0)	4 (14.8)		1 (10.0)	2 (6.7)	
Some college	3 (33.3)	11 (40.7)		3 (30.0)	9 (30.0)	

4-year college degree or more	6 (66.7)	5 (18.5)		6 (60.0)	17 (56.7)	
**Body Mass Index (kg/m^2) (Median (IQR))**	24.3 (21.8-27.3)	32.9 (27.7-38.4)	**0.003**	22.8 (21.4-27.7)	26.2 (22.5-32.0)	0.15
**Hemoglobin A1c (Median (IQR))**	6.1 (5.8-6.6)	7.5 (6.4-8.8)	**0.008**	5.5 (5.3-5.7)	5.1 (5.0-5.3)	**0.007**

[a] The Mann-Whitney test for skewed continuous variables, Fisher exact test for binary variables, and exact chi square test for other categorical variables.

P-Value < 0.05 is **bolded**

S3 Table. Periodontitis severity and clinical periodontal measures for adults with CF and non-CF controls ages 30 years and older grouped by CF-diabetes status (N=76).

**Periodontitis severity** [a]	**CFRD**<br>**N=9**<br>**N (%)**	**CF- no diabetes**<br>**N=10**<br>**N (%)**	**Non-CF with diabetes**<br>**N=27**<br>**N (%)**	**Healthy**<br>**N=30**<br>**N (%)**
No periodontitis	2 (22.2)	4 (40.0)	12 (44.4)	28 (93.3)
Mild periodontitis	2 (22.2)	4 (40.0)	1 (3.7)	2 (6.7)
Moderate periodontitis	5 (55.6)	2 (20.0)	10 (37.0)	0 (0.0)
Severe periodontitis	0 (0.0)	0 (0.0)	4 (14.8)	0 (0.0)
**Periodontal measure**	**Mean ±SD**	**Mean ±SD**	**Mean ±SD**	**Mean ±SD**
Periodontal pocket depth (mm)	2.4±0.11	2.4±0.13	1.5±0.56	1.1±0.28
Clinical attachment loss (mm)	1.8±0.32	1.7±0.28	1.7±0.97	1.2±0.28

[a] Defined following the U.S. Centers for Disease Control and Prevention and the American Academy of Periodontology case definition.

S1 Fig. Identification and enrollment of adults with CF.

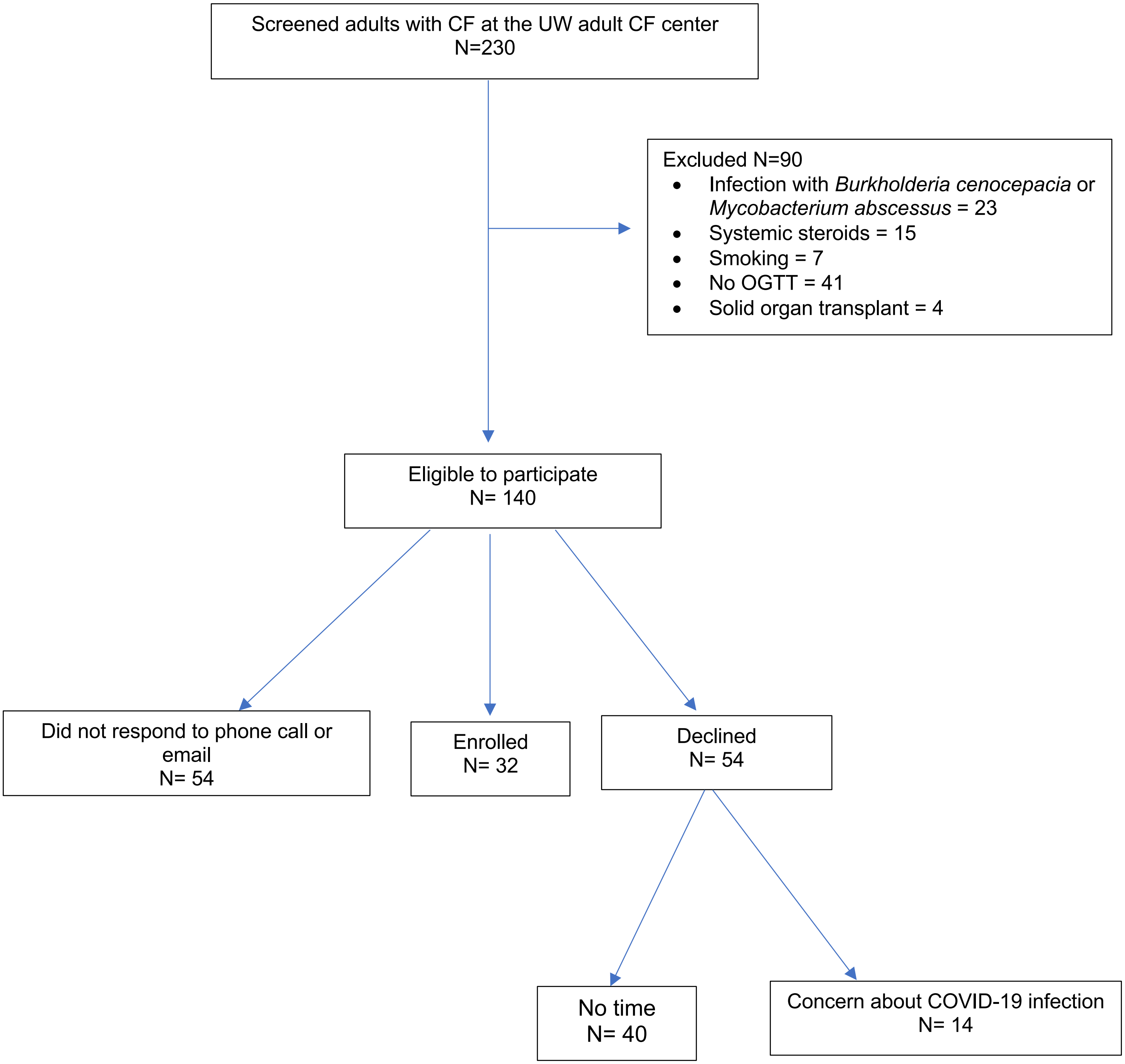

S2 Fig. Identification of non-CF controls from NHANES 2013-2014.

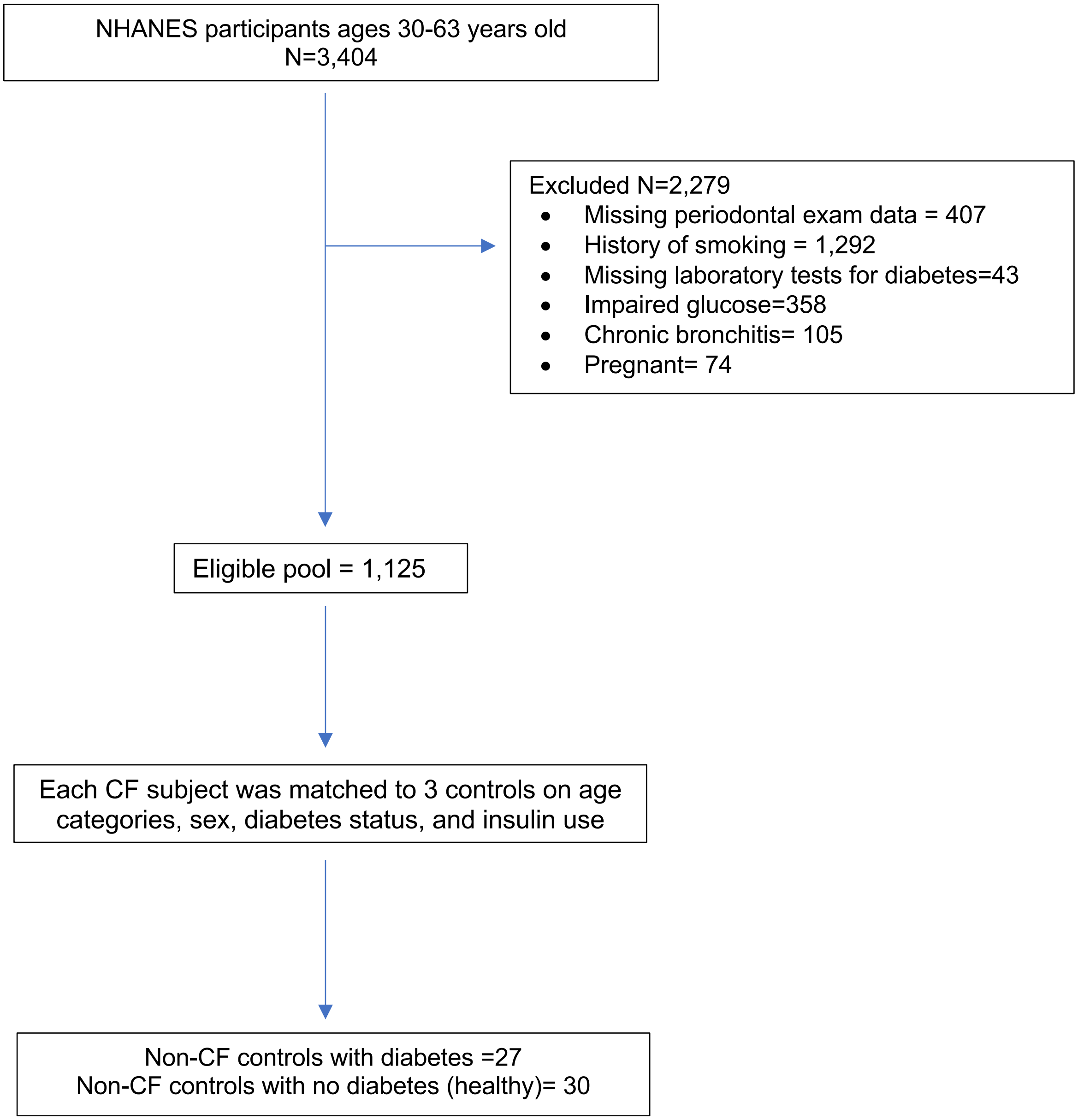

Supplement: S1 File — (PDF) [file pone.0305975.s002.pdf]
